# Fractionation and Antibacterial Evaluation of the Surface Compounds from the Leaves of *Combretum zeyheri* on Selected Pathogenic Bacteria

**DOI:** 10.1155/2023/2322068

**Published:** 2023-07-21

**Authors:** Monica Manyawi, Winnie Yevai Mozirandi, Dexter Tagwireyi, Stanley Mukanganyama

**Affiliations:** ^1^Department of Pharmacy and Pharmaceutical Sciences, Faculty of Medicine and Health Sciences, University of Zimbabwe, Mt. Pleasant, Harare, Zimbabwe; ^2^Department of Biotechnology and Biochemistry, University of Zimbabwe, Mt. Pleasant, Harare, Zimbabwe

## Abstract

*Combretum zeyheri* is traditionally used for the treatment of many infections, including bacterial infections. The aim of this study was to fractionate and evaluate antibacterial activity of the crude extract of *C. zeyheri*, as well as the surface compounds from the leaves of *C. zeyheri*, in two pathogenic bacteria, *Staphylococcus aureus* and *Pseudomonas aeruginosa*. The antibacterial activities of fractions obtained from chromatographic separations were determined using broth microdilution assay on the laboratory and clinical strains of *S. aureus* and *P. aeruginosa*. The fractionation of the compounds on the leaf surface yielded 262 fractions. The fractionated compounds with similar TLC profiles were pooled together to yield 47 pools. The extract and pooled fractions CZSC151154, CZSC155160, and CZSC209213 showed significant antibacterial activity with MIC values ranging from 12.5 *μ*g/ml to 100 *μ*g/ml. The clinical strain of *S. aureus* had MIC greater than 100 *μ*g/ml for CZSC151154 and CZSC155160. The minimum bactericidal concentration values for these fractions were also in the range of 12.5 *μ*g/ml to 100 *μ*g/ml. The extract and fractions CZSC151154, CZSC155160, and CZSC209213 showed a concentration-dependent inhibition of growth in *S. aureus*. Analyses of the CZSC209213 pool by LC-MS showed the presence of nine compounds which are (3R,7R)-1,3,7-octanetriol, (-)-tortuosamine, 11-aminoundecanoic acid, 1-piperidinecarboxaldehyde, 3-hydroxy-4-isopropylbenzyl alcohol 3-glucoside, hydroxy-isocaproic acid, oleamide, palmitic amide, phytospingosine, and sphinganine. In conclusion, *C. zeyheri* leaf surface compounds exhibited antibacterial activity. The crude extract and the pooled fractions showed concentration-dependent inhibition of growth on *S. aureus.* Results from this study indicate the potential of *C. zeyheri* as a source of lead compounds that may be further developed into antibacterial drugs.

## 1. Introduction

Zimbabwe is rich in fauna and flora; it has more than 5000 different plant species and 10% of these are believed to be of great medical importance [1]. The use of medicinal plants in Zimbabwe has been used since time immemorial for the treatment and management of various ailments. It is mostly seen in the rural population, with more than 80 percent depending on these medicinal plants. This is because medicinal plants are readily available and affordable for poor communities in rural areas [2]. However, this raises concerns about the over harvesting of these plants that will lead to the extinction of some plants [3]. A group of six organisms has recently been labelled as ESKAPE pathogens (*Enterococcus faecium*, *Staphylococcus aureus*, *Klebsiella pneumoniae*, *Acinetobacterbaumannii*, *Pseudomonas aeruginosa, and Enterobacter spp.*) [4]. *P. aeruginosa* which is among the ESKAPE pathogens is resistant to many antibiotics. It is regarded as a multidrug-resistant organism as it exhibits several drug-resistant mechanisms [5]. Resistance is exhibited through the presence of drug efflux pumps coupled together with low permeability of the outer membrane to drugs. *S. aureus* is another ESKAPE pathogen which is ubiquitous and forms part of the human microbiota and resides in the nose, inguinal areas, and also on the skin. Infection occurs when the bacteria manages to evade the protective mechanisms of the host, for example, when the host is injured [6].

Plants belonging to the Combretaceae family are the most widely used medicinal plants for traditional medicinal purposes in Africa [7]. Terminalia and Combretum species are mainly used for the management of bacterial, fungal, and viral infections; and they are also used for the treatment of bilharzia [8]. In addition to their antimicrobial properties, they possess analgesic, anticancer, anti-inflammatory, and antioxidant properties, they are also used for the management of heart disease [7]. The genus Combretum comprises 250 species; of these species, over 24 species are used for medicinal purposes [9].

*Combretum zeyheri* is found in southern parts of Kenya, DR Congo, and north-eastern parts of South Africa [10]. In Zimbabwe, it is distributed in the tropical grassland (savanna) regions [11]. The tree grows in forests, rocky hillsides, and sometimes in termite hills [11]. *C. zeyheri* has been previously documented to possess antimicrobial properties. The whole leaf extract was shown to have antifungal activity against *C. albicans* and *C. krussei* with MIC of 0.08 *μ*g/ml and 0.16 *μ*g/ml, respectively [11]. This shows that the *C. zeyheri* leaf extract contains phytochemicals that are responsible for the antimicrobial activity. These phytochemicals of *C. zeyheri* can be isolated and can serve as new chemical entities for the development of new antimicrobial drugs [12].

Plants secrete a wide range of phytochemicals on their leaf surfaces that act as allelochemicals against insects and microbes [13]. These secondary metabolites diffuse to the surface of the leaf and accumulate in the glandular trichomes or the waxy cuticle. This makes plants a good source of antimicrobial compounds [14, 15].

## 2. Materials and Methods

**Figure 1:**
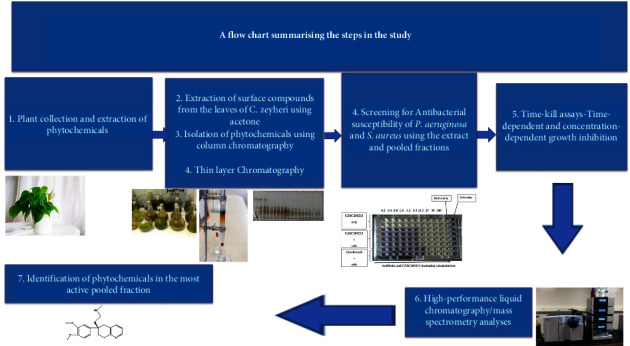
A summary diagram with the steps of this study.

An outline summarising the procedures is shown in Figure 1.

### 2.1. Chemicals and Reagents

All chemicals used in the study were obtained from Sigma-Aldrich Chemical Company (Darmstadt, Germany). All solvents used were of analytical reagent grade, and these were acetone (extractant), methanol, n-hexane, methanol, ethyl acetate, and chloroform used for column chromatography. Dimethylsulfoxide (DMSO) was used for dissolving the crude extract and fractions for assay. Silica gel mesh of size 60–120 was used for the preparation of the extract and stationary phase; filtration was performed on Whatman filter paper number 1. TLC plates were obtained from Sigma-Aldrich Chemical Co. Distilled water was collected from the Pharmacy Department. A rotary evaporator was used for fraction concentration. The strains used were laboratory strain *P. aeruginosa* (ATCC 27853), *P. aeruginosa* clinical strain, laboratory strain *S. aureus* (ATCC 9144), and *S. aureus* clinical strain. The clinical strains were procured from the Parirenyatwa Group of Hospitals from the College of Health Sciences and Department of Microbiological Sciences of the University of Zimbabwe, while the laboratory strains were from the Department of Biological Science at the University of Botswana. Determination of all antibacterial activities was carried out in a Class 2 biosafety cabinet.

### 2.2. Plant Collection and Extraction of Phytochemicals

The leaves were collected from Norton (Geographic Coordinates of Norton, Zimbabwe; latitude: 17° 52′59′ S; longitude: 30° 42′00′E; elevation above sea level: 1360 m; Mashonaland West Province of Zimbabwe). The leaves were verified by a taxonomist at the National Herbarium and Botanical Gardens. The surface compounds were extracted according to Omosa et al. [16], with modifications. The leaves were first washed under running water to remove any dust particles and then dipped in acetone for 15–30 seconds. Filtration was performed to remove any residues, and the acetone evaporated overnight under a cold stream of cold air to concentrate the extract until it was dry.

### 2.3. Thin Layer Chromatography

TLC was used to determine the mobile phase for column chromatography. TLC plates (aluminium coated with Merck, silica gel 60 F_254_) were developed under saturated conditions with solvents of different polarities, hexane:ethyl acetate 90 : 10 up to 85 : 15 and chloroform:methanol 90 : 10 up to 80 : 20. The surface compounds extracted at 100 *μ*g/ml were applied 1 cm from the bottom of the plates using a micropipette and allowed to develop in the tank saturated with an eluent. Once developed, the separated compounds were observed under UV light at 254 nm and 360 nm. Sulphuric acid was also used for visualisation, and it was sprayed and heated to 110°C. The best mobile phase was the one that showed distinct separation of compounds.

### 2.4. Isolation of Phytochemicals Using Column Chromatography

The extract was dissolved in minute amounts of methanol. It was adsorbed in an equal amount of 60–120 silica mesh and added in very small amounts until a crunchy mixture formed. This was left to dry overnight and ground to a fine powder using a mortar and pestle. Silica 60–120 which was four times the amount of the extract was mixed with 0.532 L of ethyl acetate to form slurry. The slurry was carefully poured into the length of the column (4 cm × 120 cm). The dead volume was determined and the column was left to pack overnight. The prepared extract was poured into the column and a plug of cotton wool was placed on top to prevent disturbances as the mobile phase was being poured into the column. Solvents of different polarities were slowly passed through the column in order of increasing polarity to facilitate separation. The fractions collected from each solvent polarity were concentrated using a rotary evaporator (BUCHI™ R2, Switzerland) and poured into vials that were left to dry on the bench. The purity of the fractions was determined using TLC.

### 2.5. The Effects of Fractionated Extracts on Bacteria

**Figure 2:**
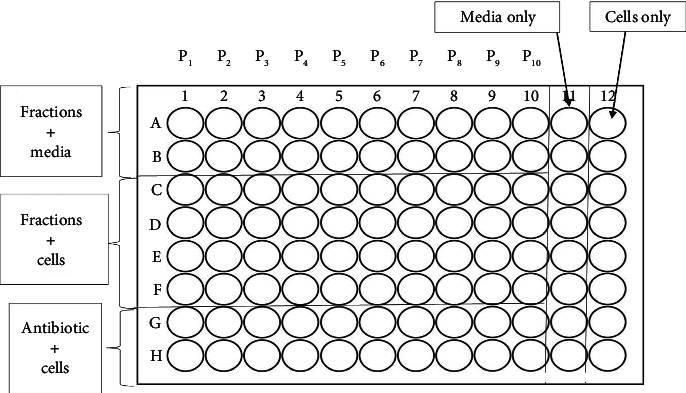
The plate layout for MIC determination of antibacterial effects on the crude extract, CZSC151154, CZSC155160, and CZSC209213, with media and bacterial cells serving as sterility controls.

Antibacterial susceptibility tests were carried out according to the broth microdilution method according to Vangeas et al. [17]. The bioactivity of the pooled fractions was screened using sterile polystyrene 96-well plates at the highest concentration of 100 *μ*g/ml (Figure 2).

The antibiotic ciprofloxacin was used as a standard drug. The number of viable bacteria in the sample used for the study was 1 × 10^6^ cfu/ml according to the CLSI standard guidelines. Each plate accommodated 10 different fractions for screening. The plate was covered with a cling wrap paper and incubated at 37°C in humid conditions for 20 hours. After 20 hours of incubation, readings were taken, and the results were analysed to give the MIC. MTT assays were carried out to serve as an indicator of growth. Yellow MTT forms insoluble purple formazan crystals in the presence of a viable cell [18]. MTT of volume 20 *μ*l of concentration 0.2 mg/ml was added to the wells and incubated for 2 hours. This assay confirmed the MIC value since MTT is an indicator of growth. The lowest concentration that showed no colour change was the MIC.

### 2.6. Determination of MBC

The most potent pooled fractions and the crude extract were chosen for MBC determination. Three fractions (CZSC151154, (24) CZSC155160 (25), and CZSC209213 (39)) that had the greatest inhibitory activities were chosen for the determination of MBC. An inoculum of bacteria from the wells with the lowest concentration to the highest concentration of the fractions showing no visible growth was streaked on the agar. Positive and negative controls were incorporated into the assay. The MBC value was the lowest concentration that showed no visible growth in the Petri dishes.

### 2.7. Time-Kill Assays

The time-dependent and concentration-dependent growth inhibition studies were carried out on *S. aureus* (ATCC) which was more susceptible to all the fractions. The effects of the different concentrations of the extract and the fractions on the growth rate of *S. aureus* were determined by using the 96-well microplate using the turbidometry method according to Banfi et al. [19], with some modifications. The bacterial inoculum (1 × 10 ^6^ CFU/ml) was exposed to varying concentrations of the extract and fractions which were ½ × MIC, 1 × MIC, and 2 × MIC, and MBC and OD readings were recorded using the Tecan microplate reader (Tecan, Genios Pro Microplate Reader, Grödig, Austria) after every 2 hours for the first 10 hours and then at 20 and 24 hours.

### 2.8. High-Performance Liquid Chromatography/Mass Spectrometry Analyses

The constituents of the most potent extract that were in the CZSC209213 pool were analysed using liquid chromatography-mass spectroscopy in the Department of Pharmacy of the University of Zimbabwe. An Agilent HPLC 1260 system (Agilent, USA) was used equipped with a binary pump, autosampler, and thermo-stated column compartment. Chromatographic separation was performed using Agilent Poroshell 120, C18 Column, with dimensions 50 × 2.7 mm, 3 microns. The mobile phase for the positive mode consisted of 0.1% formic acid in deionised water (A) and 0.1% formic acid in acetonitrile (B). The total run time for each sample was 25 min. The flow rate was 0.25 mL min^−1^, and the injection volume was 5 *μ*L. The column temperature was maintained at 40°C. An Agilent Technologies Q-TOF 6530 Mass Spectrometer was used as the detector. Agilent Technologies Mass Hunter Software version B.07.03 (509) was used for data acquisition, instrument control, and data analysis.

### 2.9. Statistical Analyses

Graphical and statistical analyses were performed using GraphPad Prism version 5. Data were expressed in the form of mean ± standard deviation of the mean. Statistically significant differences between various means of controls and tests were analysed using one-way ANOVA using Dunnett's multiple comparison posttest with a *p* value of 0.05.

## 3. Results and Discussion

Antibacterial resistance results from misuse, irrational prescribing, and poor patient compliance. Bacterial species multiply at a faster rate and have a high ability to change genetic material, making the available antibiotics ineffective [20]. The resistance forms of the pathogenic bacteria are associated with poor clinical outcomes compared with their susceptible counterparts. Poor prognosis means longer hospital stays, increased mortality, and higher hospitalisation costs [16]. The Infectious Diseases Society of America (ISDA) developed the acronym ESKAPE to emphasise the group of pathogens that cause hospital infections and effectively “escape” the effects of antibacterial drugs [17]. New antibacterial agents and plants that can be a useful source of lead compounds for drug development are needed. This study highlights the effects of surface compounds isolated from *Combretum zeyheri* leaves as sources of antibacterial agents. We previously showed the inhibitory effects of *Combretum zeyheri* leaf extracts and their S9 metabolites against *Escherichia coli*, *Bacillussubtilis*, and *Candida albicans* [24]. The plant extract also showed inhibitory effects on drug efflux in all three organisms, suggesting that the antimicrobial actions could be partially due to the inhibition of drug efflux pumps. The S9 metabolites of *C. zeyheri* also had an inhibitory effect on the growth of *C. albicans*, suggesting that they are inhibitors of the growth of *Candida albicansin vivo*.

### 3.1. Separation and Identification of Surface Compounds

A total of 262 fractions were collected from the column chromatographic separation. Fractions showing similar TLC profiles were pooled together to obtain 47 pools. Profiles for the identification of separated fractions as well as their antibacterial effect on *P. aeruginosa* and *S. aureus* are shown are Tables 1–3.

### 3.2. Comparison of the Effects of Fractionated Pools and Extracts on Bacteria

Bioactivity screening was carried out to determine the most potent pooled fractions. The extracts were screened at the highest concentration of 100 *μ*g/ml. Laboratory strains of *P. aeruginosa* and *S. aureus* were used in the study. The bacterial species were incubated overnight at a concentration of 100 *μ*g/ml of the pooled fractions. The results were plotted as a graph of cell density versus the concentration (100 *μ*g/ml) of the pooled fractions; the % effect of these pooled fractions on the inhibition of growth of the test pathogens was calculated from these graphs and is shown in Tables 1–3. All the pooled fractions significantly inhibited the growth of *P. aeruginosa*. There was a significant inhibition of growth of the bacteria for fraction CZSC2728 to pooled fraction CZSC133139 when compared with the control. There was complete inhibition of growth of the bacteria for the pooled fraction CZSC141146 to pooled fraction CZSC260261. Ciprofloxacin was used as a positive control and the MIC was 0.25 *μ*g/ml. The effects of the pooled fractions on *S. aureus* (ATCC) are also shown in Tables 1–3. CZSC2932, CZSC5051, and CZSC9596 did not significantly inhibit the growth of *S. aureus*. Fraction CZSC2728 to fraction CZSC121131 significantly inhibited the growth of *S. aureus.* Ciprofloxacin was used as the positive control drug and the MIC was 0.25 *μ*g/ml.

Since plant extracts contain a variety of phytochemicals which include alkaloids, tannins, terpenes, and saponins, they can be separated according to their varying polarities. In the study, column chromatography was utilized for separation together with the most used method of identification TLC [19]. The study showed that there was a decrease in activity after fractionation which occurred from pooled fractions CZSC2728 to CZSC133139 which had no MIC at the highest concentration of 100 *μ*g/ml used in the study. The antibacterial activity of the fraction CZSC209213 was increased and that of CZSC151154 and CZSC155160 was reduced compared to the original unfractionated extract. These results are in agreement with the trend observed in a study by Njateng et al. [21], who found that the fractionation crude extract of *Polyscias fulvaHiern (Araliaceae)* led to an increase in antibacterial activity of the fractions on the bacterial species *S. typhi*, *E. aerogenes*, *P. aeruginosa,* and *E. coli*. In a study by Nwodo et al. [22], the fractionation of the crude methanolic bark extract of *T. indica* led to an increase or reduced antibacterial activity of the fractions. After the fractionation on the column, pooled fraction CZSC209213 was found to be more active than the extract. In a study, Milugo et al. [23] reported on the antagonistic effects of phytochemicals affecting the antioxidant activity of the crude extract of *Rauvolfia caffra*. In that study, the presence of saponins and alkaloids was found to reduce the antioxidant properties of the crude extract [23].

*Combretum* species are known to have activity against both Gram-positive and Gram-negative bacteria [7, 24]. In a study by Eloff et al. [25], the antibacterial activity of the whole leaf extract of *C. zeyheri* in both *S. aureus* and *P. aeruginosa* was reported to be 0.8 mg/ml, showing that *C. zeyheri* has activity against both Gram-positive and Gram-negative bacteria. In the study, both clinical and laboratory strains were used. Clinical strains were used to represent the pathogenesis of “real life” because laboratory strains may lack some pathophysiological characteristics [26]. Laboratory strains are reference strains as required by NCCLS, and clinical strains have been exposed to selective pressures in clinical settings when patients are being treated [25]. Laboratory strains were more sensitive to surface compounds compared to clinical strains, due to the selective drug pressure to which clinical strains are exposed. In a study by Mukanganyama [27], it was reported that laboratory strains were more susceptible.

### 3.3. MIC and MBC Determination for Fractions CZSC151154, CZSC155160, and CZSC209213

**Figure 3:**
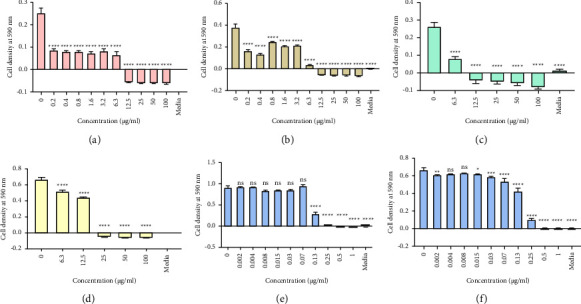
The antibacterial effect of CZSC209213 on (a) *P. aeruginosa* ATCC, (b) *P. aeruginosa* (clinical strain), (c) *S. aureus* ATCC, and (d) *S. aureus* (clinical strain). (e, f) The effect of the positive control, ciprofloxacin, on *P. aeruginosa* (clinical strain) and *S. aureus* (clinical strain), respectively.

Three pooled fractions CZSC151154, CZSC155160, and CZSC209213 that showed the highest inhibitory activities for both bacteria in initial antibacterial screening assays were chosen for the determination of MBC. The effects of the pooled fractions CZSC209213 on the growth of *P. aeruginosa* and *S. aureus* are shown in Figure 3. The data for all three fractions are summarised in Table 4. The most active pooled fraction was CZSC209213 shown in Table 4; the order of potency of the pooled fractions and the crude extract was CZSC209213 > extract > CZSC155160 > CZSC151154. The extract had MIC of 100 *μ*g/ml for *S. aureus* (clinical strain) and 25 *μ*g/ml for *S. aureus* (ATCC) and both the laboratory and clinical strains of *P. aeruginosa*. The second most potent pooled fraction of the three pooled fractions tested was CZSC155160 which had the same MIC value as the crude extract of *S. aureus* (ATCC) and both the laboratory and clinical strains of *P. aeruginosa*, but the MIC of *S. aureus* (clinical strain) was greater than 100 *μ*g/ml used. The least active pooled fraction of the three pooled fractions was CZSC151154 which had MIC of 50 *μ*g/ml for both the laboratory and clinical strains of *P. aeruginosa*, MIC of 25 *μ*g/ml for *S. aureus* (ATCC), and MIC (clinical strain) greater than 100 *μ*g/ml for *S. aureus*.

The order of potency was as follows: CZSC209213 > crude extract > CZSC155160 > CZSC151154.

For MBCs, there was a two-fold decrease in the MIC for both species of bacteria. Since MICs and MBCs were not that different between extracts and fractions, this may signify that the phytochemicals in these pools were the most abundant and were responsible for the bioactivity or the enhanced bacteriostatic and bactericidal effects observed.

### 3.4. Time-Kill Assays

**Figure 4:**
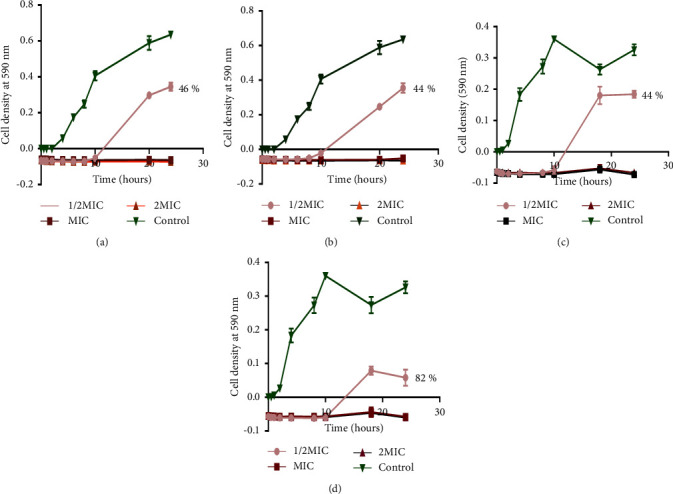
The effect of (a) the extract, (b) CZSC151154, (c) CZSC155160, and (d) CZSC209213 on the growth curve of *S. aureus*. Concentration-dependent inhibition of growth of bacteria is shown by all the 3 fractions and the extract. Prolongation of the lag phase occurred compared with the control (*S. aureus*). The duration of the lag phase for ½ MIC for (a) was 11.1 hours, (b) was 10 hours, (c) was 11.5 hours, and (d) was 12.9 hours. The % inhibition of the ½ MIC values is shown in the graphs.

The effects of the extract and the pooled fractions on the growth curve of*S. aureus* were determined using the turbidimetric method at varying concentrations. In all cases, the MIC was equivalent to the MBC. The three pooled fractions CZSC151154, CZSC155160, and CZSC209213 and the extract showed a concentration-dependent inhibition effect on *S. aureus growth* (Figure 4). In all cases, the concentrations at 1 × MIC and 2 × MIC values completely inhibited growth from time *t* = 0 to time *t* = 24. The ½ × MIC value showed that the lag phase of the bacterial growth curve was prolonged compared with the positive control. Of note was that all samples, except the control, showed a negative OD value at time zero. This essentially shows that the MIC and the 2 × MIC concentration had an immediate reduction effect on cell density compared to the control for the 24 hours of investigation. The duration of the lag phase for the positive control was 2 hours. The duration of the lag phase for ½ MIC for the extract was 11.1 hours (Figure 4(a)); for CZSC151154, it was 10 hours (Figure 4(b)), for CZSC155160, it was 11.5 hours (Figure 4(c)), and for CZSC209213, it was 12.9 hours (Figure 4(d)). The % inhibition of the ½ MIC values are shown on the graphs with CZSC209213 having the highest % inhibition of 82% (Figure 4(d)), the extract having a % inhibition of 46% (Figure 4(a)), and a % inhibition of 44% for both CZSC151154 and CZSC155160 (Figures 4(b) and 4(c), respectively).

It is expected that the Gram-negative bacteria are generally less sensitive to plant extracts and drugs due to the presence of an outer membrane which acts as a permeability barrier [7]. However, this was not observed with the clinical strains used in the study. Time-kill kinetics also provides information on the speed of bactericidal activity of a plant extract. An ideal antibacterial agent would be one that kills bacteria in a short time to prevent the bacteria from becoming resistant [18]. In the study, the high concentrations of MIC and 2 × MIC completely inhibited the growth of the bacteria and did not allow the exponential phase to be reached, and ½ × MIC value of 1 2 MIC did not completely inhibit the growth even after exposure of the bacteria for 24 hours. The bacterial growth curve consists of three phases: the lag phase during which the bacteria initiate an infection and the exponential phase. In the exponential phase, virulence factors are synthesized and there is also active growth of the bacteria and lastly the stationary phase where the bacteria produce toxins and exotoxins and develop sensing mechanisms for spreading to other tissues [28]. The ability of bacteria to initiate an infection lies in the presence of virulence factors, which are synthesized in the exponential phase. Therefore, it is important to note that the surface compounds of *C. zeyheri* prevented *S. aureus* from entering the exponential phase and therefore exhibited high antibacterial activity.

### 3.5. LC-MS Analysis of Fraction CZSC209213

LC-MS was used to identify the compounds that could contribute to the antibacterial activity of the pooled fraction CZSC209213. The results show that CZSC209213 contained a mixture of phytoconstituents of different classes. It had a phospholipid (phytosphingosine), fatty acid amides (palmitic amide and oleamide), cholesterol (sphinganine), amine (11-aminoundecanoic acid), amino acid (hydroxy-isocaproic acid), alcohol ((3R,7R)-1,3,7-octanetriol), and alkaloid ((-)-tortuosamine). The chemical formula of these compounds are provided as supplementary data at the end of the manuscript as Supplementary Table 4 and their structures are also shown in the supplementary material as Supplementary Table 5. Medicinal plant products are likely to have many variations in phytochemical content due to several contributing factors which include the plant's identity, seasonal variations, and geographical location [29]. To ensure that health as a basic need for humans is affordable and safe, the WHO stresses the importance of qualitatively and quantitatively characterizing of medicinal plant products in traditional medicinal practise [30]. LC-MS analysis of CZSC209213 led to the identification of 9 compounds belonging to different classes of phytoconstituents. The antibacterial activity of CZSC209213 can be attributed to the presence of phospholipids (phytosphingosine and sphinganine) in the pooled fraction. These phospholipids can also be found on the epidermis of the human skin and offer protective effects against pathogens [31]. Phytosphingosine was found to be antibacterial with an MIC of 200 *μ*g/ml on *S. aureus* and sphinganine with an MIC of 100 *μ*g/ml on *P. aeruginosa*. In a study by Drake et al. [32], sphinganine acted by disrupting the bacterial cell wall, resulting in invaginations of the plasma membrane and loss of ribosomes in the cytoplasm [33].

Most of the compounds identified by LC-MS from the leaves of *Combretum zeyheri* have been reported to have antibacterial effects. Phytosphingosine (PS) has been shown to be active against *Staphylococcus aureus*, the etiological cause of atopic dermatitis [34]. Phytosphingosine (PS) is a natural antimicrobial ingredient present in the stratum corneum of mammalian skin [35]. The presence of this compound in the leaves makes it an important component of the plant, thus, justifying the use of the plant in Zimbabwean traditional medical practices. In the study by Başpınar et al. [35], it was shown to have antimicrobial activity against Gram-positive and Gram-negative bacteria and *Candida* strains. It has been reported that fatty acids and their amide derivatives are natural-defence agents in plants [36]. In terms of mechanism of action, the amides target protein synthesis and cause leakage of intracellular components in the organisms. It is postulated that the presence of palmitic amide and oleamide in the surface extracts of *Combretum zeyheri* would exert antimicrobial activities. Therefore, the fatty acids and their amides can be exploited as therapeutics that can cover a wide range of indications of bacterial infections, as shown in this study. The derivatives of phospholipids (C16 sphinganine) and fatty acids (11-aminoundecanoic acid) and palmitic amide have also been isolated by LC-MS from mushrooms that have been reported to be responsible for the antibacterial activity against *Staphylococcus aureus* [37]. The compound 2-hydroxy-isocaproic acid (HICA) was shown to have antibacterial activity against obligate anaerobic bacterial species associated with periodontal disease [38]. The compound 1-piperidinecarboxaldehyde has been isolated from an herbal medicine that contains extracts of *Zingiber officinale*, *Piper longum*, and *Piper nigrum* [39]. Tortuosamine is an alkaloid found in *Sceletium tortuosum*. Traditionally, this medicinal plant is mainly masticated or smoked and is used to relieve toothache and abdominal pain, as a mood enhancer, analgesic, hypnotic, anxiolytic, thirst and hunger suppressant, and for its intoxicating/euphoric effects [40]. The compound 3-hydroxy-4-isopropylbenzyl alcohol 3-glucoside belongs to the class of organic compounds known as terpene glycosides. Terpene derivatives have been shown to be a potential agent against antimicrobial resistance (AMR) pathogens such as *A. baumannii* and *S. aureus* [41].

## 4. Conclusions

The surface compounds from the leaves of *C. zeyheri* were found to have significant antibacterial activity. After fractionation, CZSC209213 had greater antibacterial activity compared to the crude extract, and CZSC151154 and CZSC155160 had lower activity than the extract. The antibacterial activity of CZSC2728 to CZSC133139 was significantly reduced after fractionation. The extracts CZSC209213, CZSC151154, and CZSC155160 showed a concentration-dependent inhibition effect on the growth of *S. aureus*. LC-MS analyses showed the presence of phytospingosine, palmitic amide, oleamide, spinganine, 11-amino-undecanoic acid, hydroxy-isocaproic acid, 1-piperidinecarboxaldehyde, 3-hydroxy-4-isopropylbenzyl alcohol 3-glucoside, (3R,7R)-1,3,7-octanetriol, and (-)-tortuosamine. These compounds contribute to the antibacterial activity of the leaf surface compounds found in *C. zeyheri*. Further isolation of single chemical species is required to develop lead compounds with antibacterial activity.

## Figures and Tables

**Table 1:** Antibacterial activities of the fraction separated by chromatography and pooled based on the TLC profile.

Pool number	TLC fraction	Pooled fraction name	% inhibition of growth of *S. aureus*	% inhibition of growth of *P. aeruginosa*
Pool 1	27-28	CZSC2728	28	74
Pool 2	29–32	CZSC2932	—	79
Pool 3	33–36	CZSC3336	17	83
Pool 4	43-44	CZSC4344	25	76
Pool 5	46–49	CZSC4649	22	76
Pool 6	50-51	CZSC5051	—	57
Pool 7	52–57	CZSC5257	23	79
Pool 8	59–61	CZSC5961	34	71
Pool 9	67–69	CZSC6769	51	86
Pool 10	71-72	CZSC7172	51	90
Pool 11	75-76	CZSC7576	25	44
Pool 12	79–83	CZSC7983	40	53
Pool 13	84–88	CZSC8488	24	29
Pool 14	90–94	CZSC9094	11	55
Pool 15	95-96	CZSC9596	—	52
Pool 16	98–108	CZSC98108	20	59
Pool 17	109-110	CZSC109110	49	72

—, no inhibition of growth of the test pathogen. ++, complete inhibition of growth of the test pathogen.

**Table 2:** Antibacterial activities of the fraction separated by chromatography and pooled based on the TLC profile.

Pool number	TLC fraction	Pooled fraction name	% inhibition of growth of *S. aureus* MIC/MBC^L^ MIC/MBC^C^	% inhibition of growth of *P. aeruginosa* MIC/MBC^L^ MIC/MBC^C^
Pool 18	111-112	CZSC111112	51	71
Pool 19	113–120	CZSC113120	29	70
Pool 20	121–131	CZSC121131	31	67
Pool 21	133–139	CZSC133139	++	68
Pool 22	141–146	CZSC141146	++	++
Pool 23	147–149	CZSC147149	++	++
Pool 24	151–154	CZSC151154	++ (25/25) (>100/>100)	++ (50/50) (50/100)
Pool 25	155–160	CZSC155160	++ (25/25) (>100/>100)	++ (25/25) (25/50)
Pool 26	161–166	CZSC161166	++	++
Pool 27	168–172	CZSC168172	++	++
Pool 28	173-174	CZSC173174	++	++
Pool 29	175-176	CZSC175176	++	++
Pool 30	177-178	CZSC177178	++	++
Pool 31	179-180	CZSC179180	++	++
Pool 32	182-183	CZSC182183	++	++
Pool 33	186-187	CZSC186187	++	++
Pool 34	189–194	CZSC189194	++	++

—, no inhibition of growth of the test pathogen. ++, complete inhibition of growth of the test pathogen. ND, the activities of these fractions were not determined since they were in small amounts. MIC/MBC^PaL^, values for the lab strain of *P. aeruginosa* ATCC 27853. MIC/MBC^PaC^, values for the clinical strain of *P. aeruginosa*. MIC/MBC^SaL^, values for the lab strain of *S. aureus* ATCC 9144. MIC/MBC^SaC^, values for the clinical strain of *S. aureus*.

**Table 3:** Antibacterial activities of the fraction separated by chromatography and pooled based on the TLC profile.

Pool number	TLC fraction	Pooled fraction name	% inhibition of growth of *S. aureus* MIC/MBC^L^ MIC/MBC^C^	% inhibition of growth of *P. aeruginosa* MIC/MBC^L^ MIC/MBC^C^
Pool 35	197-198	CZSC197198	ND	++
Pool 36	199-200	CZSC199200	ND	++
Pool 37	203–206	CZSC203206	++	++
Pool 38	207-208	CZSC207208	++	++
Pool 39	209–213	CZSC209213	++ (12.5/12.5) (25/>100)	++ (12.5/12.5) (12.5/12.5)
Pool 40	214-215	CZSC214215	++	++
Pool 41	216–219	CZSC216219	++	++
Pool 42	221-222	CZSC221222	++	++
Pool 43	223–234	CZSC223234	++	++
Pool 44	236–241	CZSC236241	++	++
Pool 45	243-244	CZSC243244	++	++
Pool 46	246–259	CZSC246259	++	++
Pool 47	260-261	CZSC260261	++	++

—, no inhibition of growth of the test pathogen. ++, complete inhibition of growth of the test pathogen. ND, the activities of these fractions were not determined since they were in small amounts. MIC/MBC^PaL^, values for the lab strain of *P. aeruginosa* ATCC 27853. MIC/MBC^PaC^, values for the clinical strain of *P. aeruginosa*. MIC/MBC^SaL^, values for the lab strain of *S. aureus* ATCC 9144. MIC/MBC^SaC^, values for the clinical strain of *S. aureus*.

**Table 4:** MIC/MBC values of CZSC151154, CZSC155160, CZSC209213, and the crude extract on the laboratory and clinical strains of *P. aeruginosa* and *S. aureus*.

Bacteria	MIC/MBC (*μ*g/ml)			
	CZSC151154	CZSC155160	CZSC209213	Extract
*P. aeruginosa* ATCC (27853)	50/50	25/25	12.5/12.5	25/25
*P. aeruginosa* (clinical strain)	50/100	25/50	12.5/12.5	25/25
*S. aureus* ATCC (9144)	25/25	25/25	12.5/12.5	25/25
*S. aureus* (clinical strain)	>100/>100	>100/>100	25/50	100/>100

## Data Availability

The data sets used and/or analysed during the current study are available from the corresponding author on reasonable request.

## Supplementary Materials

Supplementary Materials Table 5: the identity of the compounds that contributed to the activity of CZSC2019213 and their molecular formula and mass as identified by LC-MS. Table 6: chemical components in CZSC209213 as identified by LC-MS.
