# Supplementary material for: Fractionation and Antibacterial Evaluation of the Surface Compounds from the Leaves of Combretum zeyheri on Selected Pathogenic Bacteria
Source: ScientificWorldJournal. 2023 Jul 21;2023:2322068. doi: 10.1155/2023/2322068 (PMC10382245; doi:10.1155/2023/2322068)
Supplement: Supplementary Materials — Table 5: the identity of the compounds that contributed to the activity of CZSC2019213 and their molecular formula and mass as identified by LC-MS. Table 6: chemical components in CZSC209213 as identified by LC-MS. [file 2322068.f1.pdf]

Supplementary Material

Table 4. The identity of the compounds that contributed to the activity of CZSC2019213 and their molecular formula and mass as identified by LC-MS.

**Compound name**	**Chemical formula**	**Mass**
Phytosphingosine	$C_{18}H_{39}NO_3$	317.2952
Palmitic amide	C_{16} H_{33} N O	255.2576
Oleamide	C_{18} H_{35} N O	281.2739
Spinganine	C_{18} H_{39} N O_2	301.3006
11-amino-undecanoic acid	C_{11} H_{23} N O_2	201.1741
Hydroxy-isocaproic acid	C_6 H_{12} O_3	132.0797
1-Piperidinecarboxaldehyde	C_6 H_{11} N O	113.0851
3-Hydroxy-4-isopropylbenzyl alcohol 3-glucoside	C_{16} H_{24} O_7	328.1521
(-) – Tortuosamine	C_{20} H_{26} N_2 O_2	326.1963

Table 5. Chemical components in CZSC209213 as identified by LC-MS

**Name**[1]	**Molecular Structure**
Phytosphingosine	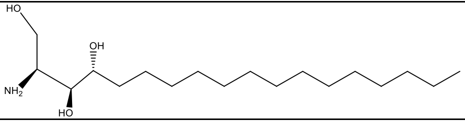
Palmitic amide	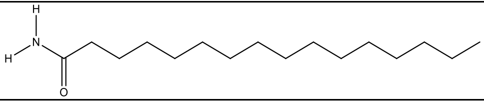
Sphinganine	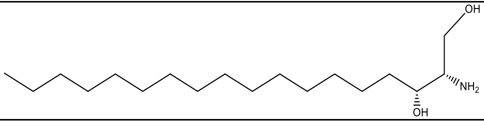
11-amino-undecanoic acid	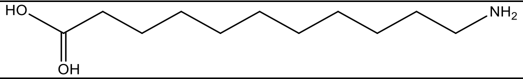
(3R,7R)-1,3,7-octanetriol	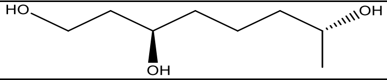
Hydroxy isocaproic acid	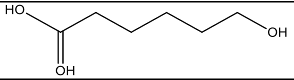

3-Hydroxy-4-isopropylbenzyl alcohol 3-glucoside	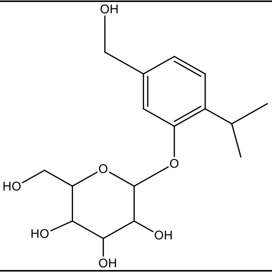
(-) - tortuosamine	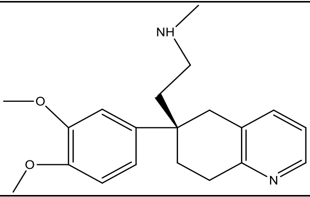
Oleamide	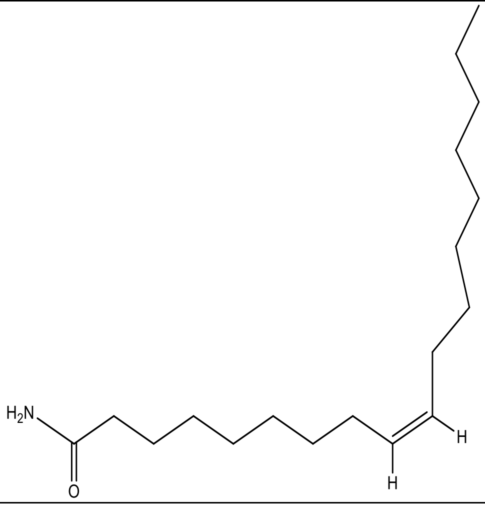

[1]The compounds phytosphingosine, palmitic amide, sphinganine, 11-amino-undecanoic acid, (3R,7R)-1,3,7-octanetriol, hydroxy isocaproic acid, 3-hydroxy-4-isopropylbenzyl alcohol 3-glucoside, (-) – tortuosamine, and oleamide were identified.

,

,
